# The Association of Hypertension With the Severity of and Mortality From the COVID-19 in the Early Stage of the Epidemic in Wuhan, China: A Multicenter Retrospective Cohort Study

**DOI:** 10.3389/fmed.2021.623608

**Published:** 2021-05-12

**Authors:** Sumaira Mubarik, Xiaoxue Liu, Ehab S. Eshak, Keyang Liu, Qing Liu, Fang Wang, Fang Shi, Haoyu Wen, Jianjun Bai, Chuanhua Yu, Jinhong Cao

**Affiliations:** ^1^Department of Epidemiology and Biostatistics, School of Health Sciences, Wuhan University, Wuhan, China; ^2^Public Health, Department of Social Medicine, Osaka University Graduate School of Medicine, Suita-shi, Osaka, Japan; ^3^Public Health and Community Medicine, Faculty of Medicine, Minia University, Minia, Egypt; ^4^Department of Epidemiology and Biostatistics, School of Public Health, Peking University, Beijing, China; ^5^Global Health Institute, Wuhan University, Wuhan, China

**Keywords:** COVID-19, hypertension, severe, mortality, critical, risk factors

## Abstract

**Background:** Hypertension may affect the prognosis of COVID-19 illness. We analyzed the epidemiological and clinical characteristics associated with the disease severity and mortality in hypertensive vs. non-hypertensive deceased COVID-19 patients.

**Methods:** We included all the deceased patients with laboratory-confirmed COVID-19 admitted to >200 health facilities in Wuhan between December 1 and February 24, 2020. The median survival time in COVID-19 patients with and without hypertension, the association of hypertension with the disease severity, and the risk factors associated with the COVID-19 mortality stratified by the hypertension status were assessed using the Kaplan-Meier survival analysis, logistic regression, and Cox proportional regression, respectively before and after the propensity score-matching (PS) for age and sex.

**Results:** The prevalence of hypertension in the studied 1,833 COVID-19 patients was 40.5%. Patients with hypertension were more likely to have severe COVID-19 illness than patients without hypertension; the PS-matched multivariable-adjusted odds ratio (95% CI) was 2.44 (1.77–3.08). Moreover, the median survival time in the hypertension group was 3–5 days shorter than the non-hypertension group. There was a 2-fold increased risk of COVID-19 mortality in the hypertension group compared with the non-hypertension group; the PS-matched multivariable-adjusted hazard ratio (HR) = 2.04 (1.61–2.72), and the significant increased risk of COVID-19 mortality in the moderate vs. mild COVID-19 illness was confined to patients with hypertension. Additionally, the history and the number of underlying chronic diseases, occupation, and residential location showed stronger associations with the COVID-19 mortality among patients with hypertension than patients without hypertension.

**Conclusion:** Hypertension was associated with the severity and mortality of COVID-19 illness.

## Introduction

In the early of December 2019, a series of sudden unfathomable cases of a respiratory disease occurred and spread rapidly among the residences in China. This disease has been named “coronavirus disease 2019 (COVID-19)” by the World Health Organization, and the coronavirus was subsequently named by the International Committee on Taxonomy of Viruses as Severe acute respiratory syndrome coronavirus 2 (SARS-COV-2) (1, 2). As of July 15, 2020, the cumulative number of confirmed cases worldwide was 12,964,863, and the cumulative death was 570,288 (3). A growing body of evidence is demonstrating that clinical comorbidities such as diabetes, hypertension, and cardiovascular diseases are highly prevalent among COVID-19 patients (4–13). Moreover, many patients with COVID-19 are critically ill and require care in the intensive care unit (ICU). Previous study suggested that the mortality rate and absolute mortality were high, hospital and ICU mortality rates were 12 and 27 per 1,000 patients-days (14). In China, the estimated mortality was 1.1% in non-severe COVID-19 patients and 32.5% in severe cases during the average 32 days of follow-up period, and severe male patients with complications may have a higher risk of death (4, 15, 16).

Hypertension was the most prevalent reported comorbidity in COVID-19 patients in Wuhan; the reported prevalence rates ranged from 15.0% (5, 9) to 36.5% (6). Suggestions were also made for some associations of the hypertension status with the severity of and mortality from the COVID-19 illness. In patients with the severe COVID-19 illness, one study reported 23.7% (5) prevalence rate of hypertension, while the rate was as high as 58.3% (10) in another study. The prevalence of hypertension was also higher in COVID-19 deceased patients; 34.0% vs. those who were discharged alive; 28.0% (7). The available systematic review and meta-analysis confirmed that hypertension was the most prevalent chronic morbidity in COVID-19 patients [17%; 95% confidence interval (CI):14–22%]. In that meta-analysis, the overall odds ratio (OR) of hypertension in patients with the severe COVID-19 illness, in reference to those with the non-severe illness, was 3.42 (95%CI: 1.88–6.22) (17). Most of the previous studies lacked the adjustment for factors that could confound the associations of hypertension with the risks of COVID-19 illness, the disease severity and the related mortality. Most importantly was the patients' age, which was suggested as the possible real contributing factor for the augmented risk among patients with hypertension, considering the high correlation between age and hypertension (7). Of the few studies that conducted multivariate adjustments, Huang et al., failed to document a significant association between the hypertension status and the COVID-19 severity or mortality in 225 patients using logistic regression models adjusted for age and sex (6). To the contrary, in a larger study which included 3,430 COVID-19 patients, of whom 1,128 had hypertension, by Zhang et al., hypertension was significantly associated with 41% higher risk of mortality due to COVID-19 after the adjustment for age, gender, and comorbid diabetes, cerebrovascular diseases, and chronic renal disease using the Cox proportional hazard regression (18).

As for the mechanism of COVID-19, the infection results in diverse symptoms and morbidity. Previous study suggests that severe COVID-19 pathophysiology includes destruction of lung epithelial cells, thrombosis, hypercoagulation, and vascular leak leading to sepsis. Specifically, COVID-19 risk factors mainly include cardiovascular disease, hypertension, and diabetes; for this population, the upregulation of the angiotensin converting enzyme-2 (ACE2) receptor is exploited by COVID-19 as the route of entry and infection. In the infection, viral envelope proteins bind to and degrade ACE2 receptors, preventing normal ACE2 function, which causes imbalances in ACE2 and induces an inflammatory immune response, known as a cytokine storm, both of which amplify comorbidities within the host (19). In the mechanism for severe COVID-19 infection, ACE2 is involved in modulating blood pressure and establishing blood pressure homeostasis (20). Several studies indicated that hypertensive patients are treated with drugs to reduce blood pressure mostly through ACE-inhibitors, that leads to increased ACE2 expression, used by the COVID-19 virus for human's cell entry (11, 21). Thus, hypertension and the severe COVID-19 infection seems to be closely associated.

To further explore the association between hypertension and the severity of the COVID-19 infection, detect the effect of hypertension on the risk of mortality in COVID-19 patients, in general, and across the different severity grades of COVID-19 infection, and to compare the other risk factors associated with the mortality in the two groups of COVID-19 patients (with and without hypertension), this study included 1,833 deceased COVID-19 patients admitted to more than 200 hospitals/community health centers in Wuhan during the early outbreak in 2019–2020.

## Methods

### Data Sources

This multicenter, retrospective cohort study was conducted based on the Chinese Infectious Disease Reporting Information System. In this system, more than 200 hospitals and community health centers have admitted almost all the COVID-19 patients in Wuhan. Data of COVID-19 patients were collected from these hospitals, including the Wuhan Hospital of Traditional Chinese and Western Medicine, Wuhan Union Hospital and Tongji, Tongji Medical College of HUST, Renmin Hospital of Wuhan University, Zhongnan Hospital of Wuhan University, Wuhan Pulmonary Hospital, Wuhan Jinyintan Hospital, Wuhan Puren Hospital, Raytheon Mountain Hospital, Airborne Military Hospital of PLA, Central Theater General Hospital of PLA, etc. All identifiable personal information was deleted for privacy protection. The epidemiological and clinical data and the hypertension status of all patients with COVID-19 were obtained from the electronic medical records of each hospital in the system. The Ethics Committee of Medical Department of Wuhan University granted ethical approval of this study (Grant number: WHU2020-2020YF0031).

### Study Population

The study population was all (*n* = 1,833) the deceased COVID-19 patients aged 18 years or more who were admitted to the designated hospitals between December 1, 2019 and February 24, 2020, with laboratory-confirmed COVID-19 infection according to the diagnostic criteria of the new coronavirus infection pneumonia diagnosis and treatment plan (trial fifth version) (22).

### Study Variables

All identifiable personal information was deleted for privacy protection. We collected the hospital admission data on the socio-demographic (age, sex, location, and occupation), and clinical characteristics including classification of the disease severity (mild, moderate, severe and critical COVID-19 illness) (18), underlying chronic disease histories (diabetes, cardiovascular disease, cerebrovascular disease, respiratory disease, and cancer). The hypertension status was ascertained via the documented medical history of the patients. Hypertension was defined as systolic blood pressure of ≥140 mmHg, diastolic blood pressure of ≥ 90 mmHg, and/or being on antihypertensive medication (18). The time-related indicators included the dates of symptoms' onset, clinical diagnosis and death, from which we calculated the durations from the symptom' onset to clinical diagnosis, and from the symptom' onset to the endpoint (death).

### Statistical Analysis

**Figure 1:**
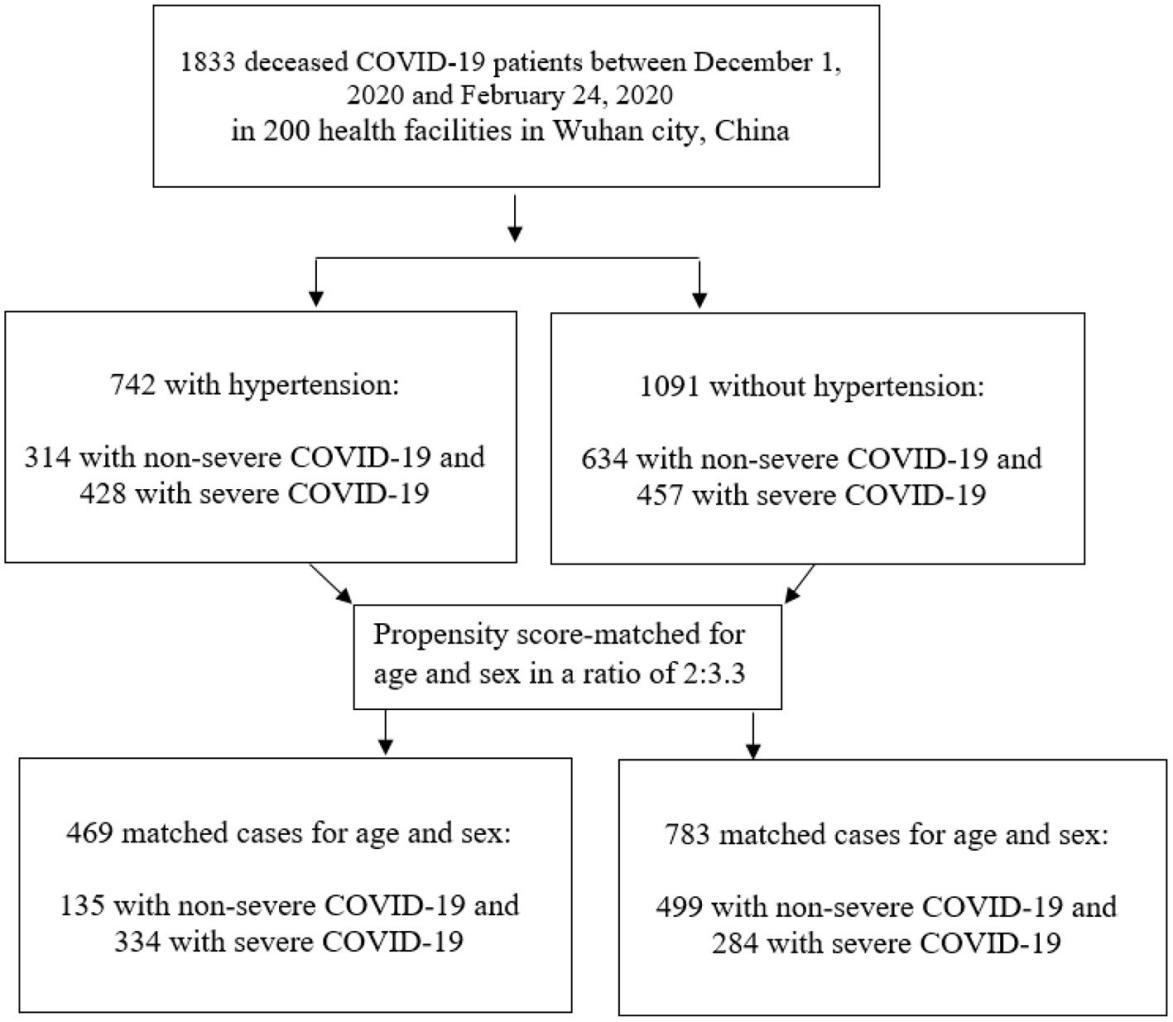
Study flow chart.

We conducted the statistical analyses twice, before and after propensity score matching for the patients' age and sex between patients in the two groups (with and without hypertension). Among total deceased COVID-19 patients, the patients with-hypertension were statistically matched (2:3.3) with patients without-hypertension according to propensity score matching without replacement (Figure 1). The logistic regression method was used to generate propensity score and matching was performed using the nearest neighbor algorithm with a caliper distance of 0.25. In practice, a wide variety of calipers is used and some studies recommended reducing the caliper from 0.25 standard deviations to 0.2 standard deviations to get the balanced groups (23–26). Previously, a caliper of 0.25 standard deviations based on the results of Cochran and Rubin (27) has been taken as a recommendation. A standardized mean difference, defined as the mean difference between the groups divided by the standard deviation of the control group was reported before and after propensity score matching (28). The variables with missing data were not included in analysis.

Further, the Kolmogorov-Smirnov test was used to test the distribution of the characteristics' variables. Continuous variables were expressed as medians and interquartile ranges (IQR), and categorical variables were described as the frequencies and percentages. The Mann-Whitney *U*-test was used to test the difference in the continuous variables and the Chi-square its Fisher exact tests were used to test the difference in the categorical variables between the two groups of deceased patients (with and without hypertension). The logistic regression analysis was used to estimate the ORs and the respective 95% CIs of hypertension in the moderate, severe, and critical COVID-19 illnesses in reference to the mild illness, and in the severe (severe/critical) vs. the non-severe (mild/moderate) groups of the COVID-19 illness. For total, non-severe, and severe COVID-19 groups, we used the Kaplan-Meier estimator to visualize the survival curves and to calculate the overall and the specific median survival times and their 95% CIs in total deceased patients and in each comorbidity, and compared them in patients with and without hypertension.

The Cox proportional hazard regression models adjusted for age, and sex in the unmatched data and matched for age and sex in the propensity score-matched data and adjusted for the other demographic and clinical factors were used to estimate the hazard ratios (HRs) and the respective 95% CIs of mortality from COVID-19 infection associated with the hypertension status among the total COVID-19 deceased patients, and among patients with different COVID-19 severity. Moreover, Cox proportional regression analyses, stratified by the hypertension status, were conducted to test the factors associated with the mortality and to compare the magnitude of the association in each factor among COVID-19 patients with and without hypertension before and after the propensity score-matching. The *p*-interaction between the hypertension status and each of the tested factors was estimated by adding a cross-product term of the dichotomous hypertension status and the target variable into the model. For all the Cox analyses, the time of follow-up was defined as the duration from the onset of symptoms to death with no censoring. There was no evidence of violations of the Cox proportional hazard assumptions as the *p*-value of the Schoenfeld residuals test were > 0.1 for all the used models. A two-sided *p* < 0.05 was considered statistically significant. All statistical analyses were performed using the SPSS (version 24.0).

## Results

### Characteristics of COVID-19 Patients With and Without Hypertension

Among the 1,833 patients diagnosed with and died from the COVID-19 illness in more than 200 health facilities in Wuhan, China between December 1, and February 24, 2020, and 742 (40.5%) patients had hypertension (Table 1). Of the total 1,833 patients, 1,211 (66.10%) were men, and the median age was 73 years (IQR, 66–80) in patients with hypertension patients and 72 years (IQR, 64–78) years in patients without hypertension. After the propensity score-matching for age and sex, the median age of COVID-19 patients was 71 years in the both the hypertension and non-hypertension groups. As for occupation, the frequency of retirees (57.40 vs. 44.30%) and housework and unemployment (20.50 vs. 15.80%) was significantly higher in patients with hypertension than patients without hypertension. When considering the geographical distribution, most cases were concentrated on central area in Wuhan, and there was no significant difference between the hypertension and non-hypertension groups. Notably, deceased COVID-19 patients with hypertension were more likely to have other comorbidities than those without hypertension, with an approximately double-fold increment for the presence of two or less other comorbidities, and almost a 7-fold increment for the presence of more than two other comorbidities among the hypertension group compared with the non-hypertension group, even after the propensity score-matching for the patients' age and sex. During the study period, the large bulk of deaths has occurred between February 1, and February 15, 2020, with more deaths in the hypertension group than in the non-hypertension group. We also observed that the median duration from onset to endpoint (death) was 15 days (IQR, 10–21) in the hypertension group and 17 days (IQR, 11–23) in the non-hypertension group; *p* = 0.047. The COVID-19 illness was critical in over a quarter of the COVID-19 patients with hypertension, while it represented not more than one-eighth of the non-hypertension group (Table 1).

**Table 1:** The epidemiological, clinical, and social characteristics of deceased COVID-19 patients at the early stage of the epidemic in Wuhan, China (Overall and stratified by the patients' hypertensive status) before and after propensity score-matching for age and sex.

**Parameters**	**Unmatched (before propensity score-matching)**					**Matched (after propensity score-matching)**				
	**All patients** ** (*n* = 1,833)**	**Patients without** ** hypertension** ** (*n* = 1,091)**	**Patients with ** **hypertension** ** (*n* = 742)**	*p*-value^*^	Standardized^****^ difference	**All patients** ** (*n* = 1,252)**	**Patients without** ** hypertension** ** (*n* = 783)**	**Patients with** ** hypertension** ** (*n* = 469)**	*p*-value^*^	Standardized^****^ difference
Age, years M(IQR)	70 (63,79)	72 (64,78)	73 (66,80)	0.56	0.10	71 (64,81)	71 (65,81)	71 (63,80)	0.16	0.01
**Gender**										
Male	1,211 (66.10)	706 (64.70)	505 (68.1)	0.13	0.17	799 (63.80)	498 (63.60)	301 (64.20)	0.84	0.07
Female	622 (33.90)	385 (35.30)	237 (31.90)			453 (36.20)	285 (36.40)	168 (35.8)		
**Occupation**										
Retirees	858 (46.80)	462 (42.30)	396 (53.40)	<0.001	0.14	616 (49.20)	347 (44.30)	269 (57.40)	<0.001	0.04
Housework and unemployment	324 (17.70)	191 (17.50)	133 (17.90)			220 (17.60)	124 (15.80)	96 (20.50)		
Public servant	25 (1.40)	21 (1.90)	4 (0.50)			12 (1.00)	10 (1.30)	2 (0.40)		
Laborers	23 (1.30)	13(1.20)	10 (1.30)			13 (1.00)	7 (0.90)	6 (1.30)		
Cadres	35 (1.90)	24(2.20)	11 (1.50)			22 (1.80)	13 (1.70)	9 (1.90)		
Farmers and migrant workers	66 (3.60)	52 (4.80)	14 (1.90)			46 (3.70)	34 (4.30)	12 (2.60)		
Medical workers	21 (1.10)	14 (1.30)	7 (0.90)			14 (1.10)	8 (1.00)	6 (1.30)		
Other occupations	481 (26.20)	314 (28.80)	167 (22.50)			309(24.70)	240 (30.70)	69 (14.70)		
**Location**										
Central area in Wuhan	1,385 (75.60)	818 (75.00)	567 (76.40)	0.08	0.16	922 (73.60)	569 (72.70)	353 (75.30)	0.23	0.10
Sub urban area in Wuhan	288 (15.70)	186 (17.00)	102 (13.70)			220 (17.60)	137 (17.50)	83 (17.70)		
Out of city/other	160 (8.70)	87 (8.00)	73 (9.80)			110 (8.80)	77 (9.80)	33 (7.00)		
**Clinical characteristics**										
Diabetes	357 (19.50)	117 (10.70)	240 (32.30)	<0.001	0.13	254 (20.30)	107 (13.70)	147 (31.30)	<0.001	0.04
Cardiovascular diseases	330 (18.00)	114 (10.40)	216 (29.10)	<0.001	0.15	251 (20.00)	107 (13.70)	144 (30.70)	<0.001	0.03
Cerebrovascular diseases	174 (9.50)	51 (4.70)	123 (16.60)	<0.001	0.04	140 (11.20)	57 (7.30)	83 (17.70)	<0.001	0.08
Respiratory	338 (18.40)	142 (13.00)	196 (26.40)	<0.001	0.26	268 (21.40)	148 (18.90)	120 (25.60)	0.005	0.04
Cancer	343 (18.70)	150 (13.70)	193 (26.00)	<0.001	0.11	266 (21.20)	153 (19.50)	113 (24.10)	0.041	0.07
Other diseases^**^	590 (32.20)	288 (26.40)	302 (40.70)	<0.001	0.10	447 (35.70)	262 (33.50)	185 (39.40)	0.032	0.05
**# of underlying disease**										
None	629 (34.30)	629 (57.70)	0 (0.00)	<0.001	0.04	373 (29.80)	373 (47.60)	0 (0.00)	<0.001	0.09
≤ 2	889 (48.50)	403 (36.90)	486 (65.50)			639 (51.00)	334 (42.70)	305 (65.00)		
>2	315 (17.20)	59 (5.40)	256 (34.50)			240 (19.20)	76 (9.70)	164 (35.00)		
COVID-19 severity^***^										
Mild	597 (32.60)	418 (38.30)	179 (24.10)	<0.001	0.06	387 (30.90)	267 (34.10)	120 (25.60)	<0.001	0.04
Moderate	351 (19.10)	21 6(19.80)	135 (18.20)			247 (19.70)	232 (29.60)	15 (3.20)		
Severe	551 (30.10)	320 (29.30)	231 (31.10)			390 (31.20)	211 (26.90)	179 (38.20)		
Critical	334 (18.20)	137 (12.60)	197 (26.50)			228 (18.20)	73 (9.30)	155 (33.00)		
**Date of onset**										
Dec-1 to Dec-31-2019	33 (1.80)	20 (1.80)	13 (1.80)	0.08	0.19	21 (1.70)	15 (1.90)	6 (1.30)	<0.001	0.06
Jan-1 to Jan-15-2020	272 (14.80)	161 (14.80)	111 (150)			184(14.70)	131 (16.70)	53 (11.30)		
Jan-16 to Jan-31-2020	1,159 (63.20)	672 (61.60)	487 (65.60)			787(62.90)	450 (57.50)	337 (71.90)		
Feb-1 to Feb-15-2020	346 (18.90)	219 (20.10)	127 (17.10)			244 (19.50)	173 (22.10)	71 (15.10)		
Feb-16 to Feb-24-2020	23 (1.30)	19 (1.70)	4 (0.50)			16 (1.30)	14 (1.80)	2 (0.40)		
**Date of diagnosis**										
Dec-1 to Dec-31-2019	3 (0.20)	3 (0.30)	0 (0.00)	0.001	0.08	3 (0.20)	3 (0.40)	0 (0.00)	0.001	0.05
Jan-1 to Jan-15-2020	32 (1.70)	24 (2.20)	8 (1.10)			22 (1.8 0)	18 (2.30)	4 (0.90)		
Jan-16 to Jan-31-2020	509 (27.80)	288 (26.40)	221 (29.80)			254 (28.30)	229 (29.20)	125 (26.70)		
Feb-1 to Feb-15-2020	1157 (63.10)	679 (62.20)	478 (64.40)			788 (62.90)	466 (59.50)	322 (68.70)		
Feb-16 to Feb-24-2020	132 (7.20)	97 (8.90)	35 (4.70)			85 (6.80)	67 (8.60)	18 (3.80)		
**Date of death**										
Jan-1 to Jan-15-2020	1 (0.10)	1 (0.10)	0 (0.00)	<0.001	0.05	0 (0.00)	0 (0.00)	0 (0.00)	<0.001	0.03
Jan-16 to Jan-31-2020	223 (12.20)	144 (13.20)	79 (10.60)			158 (12.60)	119 (15.2)	39 (8.30)		
Feb-1 to Feb-15-2020	1,229 (67.00)	662 (60.70)	567 (76.40)			844 (67.40)	470 (60.00)	374 (79.70)		
Feb-16 to Feb-24-2020	380 (20.70)	284 (26)	96 (12.90)			250 (20.00)	194 (24.80)	56 (11.90)		
Duration from onset to diagnosis, Median (IQR)	10 (6, 14)	10 (6, 15)	10 (7, 14)	0.47	0.22	10 (6, 14)	10 (6, 14)	10 (7, 14)	0.85	0.10
Duration from onset to endpoint (death), Median (IQR)	17 (12, 22)	17 (12, 23)	15 (10, 21)	0.031	0.02	17 (11, 22)	17 (11, 23)	15 (10, 21)	0.047	0.03

* *The Mann Whitney U-test was used to test the difference in the continuous variables and the Chi square or Fisher exact test was used to test the difference in the categorical variables between the two groups (with and without hypertension)*.

** *Other diseases included: anemia, hypothyroidism, Parkinson's disease, prostatic hyperplasia, fractures, etc*.

*** *The severity categories were according to the diagnostic criteria of the new coronavirus infection pneumonia diagnosis and treatment plan (trial fifth version)*.

**** *The standardized mean difference, defined as the mean difference between the groups divided by the standard deviation of the control (without hypertension) group*.

Furthermore, hypertension was associated with the COVID-19 severity in both the unmatched and matched analyses; the age-and sex-matched multivariate-adjusted ORs (95%CIs) of hypertension in patients with moderate, severe and critical COVID-19 illness in reference to that in mild illness were 2.60 (2.32–3.40), 10.60 (6.10–17.31), and 35.02 (20.11–81.00); *p*-trend < 0.001; while the OR in severe (severe and critical) in reference to non-severe (mild and moderate) illnesses was 2.44 [95% CI: 1.77–3.08], matched for the age and sex, and adjusted for occupation, location, and the number of underlying diseases (Table 2).

**Table 2:** The association between hypertension and the COVID-19 severity in the deceased COVID-19 patients using the logistic regression analysis before and after the propensity score-matching^a^.

	**Unmatched**				**Matched**			
	**Cases with** ** hypertension/** **all cases**	**Univariate OR** ** (95% CI)**	**Multivariate OR** (95% CI)^*^	**Multivariate OR** (95% CI)^**^	**Cases with** ** hypertension/** **all cases**	**Univariate OR** ** (95% CI)**	**Multivariate OR** (95% CI)^*^	**Multivariate OR** (95% CI)^**^
Mild COVID-19 illness	179/597	1.00 (reference)	1.00 (reference)	1.00 (reference)	120/387	1.00 (reference)	1.00 (reference)	1.00 (reference)
Moderate COVID-19 illness	135/351	2.62 (1.50–2.77)	3.40 (2.23–5.14)	2.80 (2.10–4.04)	15/247	1.02 (0.51–2.87)	1.14 (1.12–5.14)	2.60 (2.32–3.40)
Severe COVID-19 illness	231/551	4.72 (3.60–10.85)	9.19 (5.29–15.96)	11.66 (6.19–16.31)	179/390	2.72 (2.10–2.85)	9.13 (5.21–13.26)	10.60 (6.10–17.31)
Critical COVID-19 illness	197/334	9.44 (5.16–23.79)	42.76 (20.53–89.03)	36.16 (23.13–67.00)	155/228	2.64 (2.26–3.09)	32.16 (23.43–79.04)	35.02 (20.11–81.00)
*P*-trend		<0.001	<0.001	<0.001		<0.001	<0.001	<0.001
Non-severe COVID-19 illness^***^	314/948	1.00 (reference)	1.00 (reference)	1.00 (reference)	135/634	1.00 (reference)	1.00 (reference)	1.00 (reference)
Severe COVID-19 illness^***^	428/885	2.93 (2.62–3.04)	3.02 (2.56–3.10)	2.90 (2.00–3.10)	334/618	2.51 (2.17–2.91)	2.19 (2.09–2.76)	2.44 (1.77–3.08)

a *The adjustment for age and sex was conducted in all the multivariate models for the unmatched data, and the matching for age and sex was conducted in all the models for the matched analyses*.

* *The estimated ORs (95% CIs) of hypertension after further adjustment for occupation and location*.

** *The estimated ORs (95% CI) of hypertension after further adjustment for occupation, location and the other underlying diseases*.

*** *The non-severe COVID-19 illness included mild and moderate illnesses, while the severe COVOID-19 illness included severe and critical illnesses*.

### Kaplan-Meier Survival Analysis in COVID-19 Patients With and Without Hypertension

The log-rank test results indicated a statistically significant shorter median (95%CI) survival time in patients with hypertension than those without hypertension (Supplementary Figure 1). The overall median (95% CI) survival time was 17.0 (16.4–17.6) days in the hypertension group, while it was 20.0 (19.1–22.9) days in the non-hypertension group. The respective median survival durations were 19.0 (18.1–19.9) vs. 20.0 (16.1–22.4) for the non-severe illness and 15.0 (14.1–15.9) vs. 17.0 (16.1–17.9) for the severe illness. The curves for the patients with each specified comorbidity showed 3–4 days shorter median survival times among patients with hypertension than among those without hypertension, for accompanying diabetes, cardiovascular disease, respiratory disease or cancer; with the exception of a longer median survival time for the accompanying cerebrovascular disease in patients with hypertension than those without hypertension. The same trends were found in the stratified analyses by the COVID-19 severity; however, the largest difference was observed for the severe illness, and was the 5 days shorter median survival time in the group of hypertension with cardiovascular disease; 11.0 (8.0–14.0) than that in the non-hypertension group with cardiovascular disease; 16.0 (13.0–19.0); *P* for Log-rank test = 0.01.

### The Magnitude of the Association Between Hypertension and the Risk of COVID-19 Mortality in the Different Grades of Illness

Table 3 shows a double-fold higher risk of COVID-19 mortality in patients with hypertension in reference to those without hypertension; HR (95% CI) was 2.01 (1.79–3.13) after adjusting for age, sex, and other demographic and clinical characteristics including the severity of COVID-19 illness, and similar findings were observed in the propensity score-matched models; HR= 2.04 (1.61–2.72). The higher risk of mortality in patients with hypertension in reference to that in patients without hypertension was statistically significant and was almost of the same magnitude (3.0- to 3.7- fold) in patients with moderate, severe and critical illnesses, but was less and statistically insignificant, in case of the mild COVID-19 illness; HR (95% CI) was 2.77 (0.15–3.01) in the unmatched and 1.31 (0.11–2.21) in the matched analyses.

**Table 3:** Cox regression analysis of the association between hypertension and mortality in total COVID-19 patients and in different disease severities before and after the propensity score-matching^a^.

	**Unmatched**					**Matched**				
	**Person-days**	**Cases,** ***n***	**Univariate HR** ** (95% CI)**	**Multivariate HR** (95% CI)^*^	**Multivariate HR** (95% CI)**	**Person-days**	**Cases,** ***n***	**Univariate HR** ** (95% CI)**	**Multivariate HR** (95% CI)^*^	**Multivariate HR** (95% CI)^**^
Mild COVID-19 illness	10,445	597	2.97 (2.12, 3.16)	2.49 (0.82,2.99)	2.77 (0.15, 3.01)	6,776	387	1.24 (1.21, 2.01)	1.49 (0.23, 2.89)	1.31 (0.11, 2.21)
Moderate COVID-19 illness	6,082	351	2.85 (2.68, 3.05)	2.24 (0.61, 2.98)	3.73 (2.55, 4.95)	4,181	247	1.92 (1.51, 2.20)	2.04 (0.71, 2.54)	2.72 (2.34, 3.95)
Severe COVID-19 illness	9,955	551	4.03 (3.87, 5.22)	3.99 (3.43, 5.18)	3.61 (2.99, 4.84)	6,942	390	3.07 (3.02, 4.16)	3.68 (3.44, 4.98)	3.21 (2.89, 4.01)
Critical COVID-19 illness	5,934	334	3.18 (2.95, 4.47)	2.10 (1.87, 3.40)	3.00 (2.76, 4.32)	3,924	228	2.13 (2.10, 3.54)	2.51 (1.87, 2.66)	3.01 (2.86, 3.56)
Total COVID-19 illness of any severity	32,416	1,833	2.73 (1.93, 2.96)	1.99 (1.80, 2.09)	2.01 (1.79, 3.13)	21,823	1,252	2.62 (1.83, 2.76)	1.90 (1.73, 2.47)	2.04 (1.61, 2.72)

a *The adjustment for age and sex was conducted in all the multivariate models for the unmatched data, and the matching for age and sex was conducted in all the models for the matched analyses*.

* *The estimated mortality HRs (95% CIs) for patients with hypertension in reference to those without hypertension after further adjustment for occupation and location*.

** *The estimated mortality HRs (95% CIs) for patients with hypertension in reference to those without hypertension after further adjustment for occupation, location and the other underlying diseases*.

*** *The non-severe COVID-19 illness included mild and moderate illnesses, while the severe COVOID-19 illness included severe and critical illnesses*.

### Risk Factors for COVID-19 Mortality Stratified by the Hypertension Status

Although the demographic and clinical risk factors of mortality in COVID-19 patients were the same in the hypertension and non-hypertension groups. However, it was obvious that the risk estimates were larger in the hypertension group. The advanced age and male gender were associated with the risk in the unmatched data in both the hypertension and the non-hypertension group (Table 4). However, we repeated these stratified analyses after matching for patients' age and sex of in those with and without hypertension, by the propensity score, in Table 5, and documented similar augmented associations in the hypertension group more than that in the non-hypertension group for the suburban location in Wuhan, chronic comorbidities and the number of underlying diseases. In this stratified analysis, considering the mild COVID-19 illness as the reference, the moderate illness was associated with the higher risk of mortality only among the patients with hypertension; HR = 1.15 (1.10–1.69) but in patients without hypertension; HR = 1.01 (0.02–1.06). The mortality risk estimates for the severe and critical COVID-19 illnesses in patients with hypertension (*p*-trend = < 0.001) were approximately double those in patients without hypertension (*p*-trend = 0.002) (Table 5).

**Table 4:** Multivariable Cox regression analysis for factors associated with the mortality in 1,833 COVID-19 patients stratified by the hypertension status before the propensity score-matching for age and sex.

	**Patients without hypertension**, ***n*** **=** **1,091**			**Patients with hypertension**, ***n*** **=** **742**		
	**Person-days**	**Cases, *n***	Multivariate HR (95% CI)^*^	**Person-days**	**Cases, *n***	Multivariate HR (95% CI)^*^
**Age group (Ref:** **≤40 years)**						
Age (41–60)	4,229	221	1.02 (1.06, 2.37)	1,431	82	1.32 (1.62, 1.99)
Age (61–80)	11,749	661	1.08 (1.04, 1.60)	8,673	476	1.12 (1.04, 1.16)
Age (>80)	2,765	177	1.36 (1.26, 2.05)	2,938	181	1.92 (1.61, 2.05)
*P*-trend			0.034			0.013
**Gender (Ref: female)**						
Male	12,551	385	1.99 (1.87, 2.10)	905	505	2.99 (1.87, 3.12)
**Occupation (Ref: other)**						
Retirees	8,075	462	5.26 (2.14, 11.33)	6,897	396	7.56 (2.14, 9.93)
Housework and unemployment	3,631	191	4.18 (2.02, 12.06)	2,301	133	5.38 (2.32, 11.26)
Public servant	354	21	1.01 (0.01, 5.60)	38	4	1.03 (0.01, 6.80)
Laborers	209	13	2.38 (0.51, 8.13)	174	10	2.18 (0.13, 5.12)
Cadres	450	24	1.48 (1.73, 10.62)	187	11	1.78 (1.63, 9.61)
Farmers and migrant workers	871	52	3.11 (3.02, 12.75)	163	14	4.51 (2.23, 14.75)
Medical workers	242	14	2.07 (0.74, 11.36)	147	7	1.02 (0.34, 9.26)
**Location (Ref: Central Wuhan)**						
Sub urban area in Wuhan	3,278	186	13.21 (11.32, 21.23)	1,897	102	16.41(13.67, 26.95)
Out of city/other	1,526	87	4.06 (0.36, 4.43)	1,353	73	3.32 (0.37, 5.08)
**Chronic diseases (Ref: No)**						
Diabetes	2,107	117	3.25 (2.85, 4.60)	4,077	240	4.68 (2.57, 5.80)
Cardiovascular diseases	2,113	114	5.24 (4.01, 7.11)	3,892	216	7.17 (5.14, 8.22)
Cerebrovascular diseases	861	51	2.35 (1.70, 2.99)	2,117	123	2.88 (2.33, 3.89)
Respiratory diseases	2,643	142	5.08 (4.02, 6.37)	3,627	196	4.73 (4.12, 7.52)
Cancer	2,672	150	5.82 (5.62, 7.90)	3,632	193	6.10 (5.99, 6.42)
**Disease severity (Ref: Mild)**						
Moderate COVID-19 illness	3,648	216	1.01 (0.02, 1.06)	2,434	135	1.15 (1.10, 1.69)
Severe COVID-19 illness	5,836	320	1.28 (1.11, 1.67)	4,119	231	2.32 (1.92, 2.61)
Critical COVID-19 illness	2,557	137	1.10 (1.02, 1.70)	3,377	197	2.62 (2.22, 2.81)
*P*-trend			<0.001			<0.001
**Disease severity (Ref: non- severe)**						
Severe COVID-19	8,393	457	1.59 (1.40, 1.78)	7,496	428	2.59 (2.32, 3.02)
**Number of underlying diseases (Ref: None)**						
Number of under lying diseases ≤ 2	7,039	403	3.30 (1.06, 3.62)	8,575	486	4.21 (2.06, 5.91)
Number of under lying diseases > 2	1,113	59	2.58 (2.07, 3.91)	4,522	256	3.31 (2.81, 3.76)

* *The model included all the variables in the table*.

*The p-value for the interaction between the hypertension and various risk factors toward the risk of COVID-19 death were (0.031) for age group, (0.51) for gender, (0.64) for occupation, (0.05) for location, (0.004) for chronic diseases, (0.042) for the disease severity (4 categories), (0.040) for the disease severity (two categories), and (0.021) for the number of underlying diseases*.

**Table 5:** Multivariable Cox regression analysis for factors associated with the mortality in 1,252 COVID-19 patients stratified by the hypertension status after the propensity score-matching for age and sex.

	**Patients without hypertension**, ***n*** **=** **783**			**Patients with hypertension**, ***n*** **=** **469**		
	**Person-days**	**Cases, *n***	Multivariate HR (95% CI)^*^	**Person-days**	**Cases, *n***	Multivariate HR (95% CI)^*^
**Occupation (Ref: other)**						
Retirees	6,100	347	6.21 (3.22, 10.41)	4,626	269	6.45 (3.34, 10.33)
Housework and unemployment	2,229	124	5.12 (2.00, 11.07)	1,688	96	4.88 (3.32, 14.36)
Public servant	181	10	1.03 (0.01, 4.30)	20	02	2.05 (0.67, 7.30)
Laborers	91	07	2.48 (0.41, 7.00)	113	06	2.38 (0.13, 6.12)
Cadres	241	13	1.28 (1.13, 9.22)	148	09	1.68 (1.53, 9.71)
Farmers and migrant workers	601	34	3.01 (2.94, 11.15)	149	12	4.11 (2.43, 13.55)
Medical workers	109	08	2.36 (0.44, 13.66)	128	06	1.08 (0.64, 10.96)
**Location (Ref: Central Wuhan)**						
Sub urban area in Wuhan	2,427	137	12.31 (10.22, 20.33)	1,521	83	14.11 (12.17, 22.45)
Out of city/other	1,340	77	6.84 (0.76, 8.44)	589	33	5.62 (0.47, 7.04)
**Chronic diseases (Ref: No)**						
Diabetes	1,813	107	3.15 (2.65, 3.72)	2,455	147	3.68 (2.45, 4.91)
Cardiovascular diseases	1,892	107	4.94 (3.21, 7.11)	2,521	144	6.10 (4.74, 7.04)
Cerebrovascular diseases	977	57	2.04 (1.37, 2.83)	1,427	83	2.63 (2.17, 3.93)
Respiratory diseases	2,665	148	5.58 (3.13, 7.71)	2,138	120	4.43 (3.35, 6.76)
Cancer	2,661	153	5.11 (4.62, 8.01)	2,069	113	6.33 (4.89, 7.42)
**Disease severity (Ref: Mild)**						
Moderate COVID-19 illness	3,928	232	1.01 (0.02, 1.06)	253	15	1.15 (1.10, 1.69)
Severe COVID-19 illness	3,764	211	1.44 (1.16, 2.07)	3,178	179	2.41 (2.10, 2.82)
Critical COVID-19 illness	1,335	73	1.23 (1.14, 1.68)	2,589	155	2.52 (2.29, 3.04)
*P*-trend			0.002			<0.001
**Disease severity (Ref: non- severe)**						
Severe COVID-19	5,099	284	1.19 (1.10, 1.62)	5,767	334	2.39 (2.12, 2.91)
**Number of underlying diseases (Ref: None)**						
Number of under lying diseases ≤ 2	5,758	334	3.21 (1.30, 3.71)	5,225	305	3.52 (2.15, 5.61)
Number of under lying diseases > 2	1,352	76	2.68 (2.16, 3.43)	2,856	164	3.26 (2.69, 3.50)

* *The model included all the variables in the table*.

*The p-value for the interaction between the hypertension and various risk factors toward the risk of COVID-19 death were (0.74) for occupation, (0.12) for location, (0.013) for chronic diseases, (0.022) for the disease severity (4 categories), (0.030) for the disease severity (two categories), and (0.011) for the number of underlying diseases*.

## Discussion

The previous studies had found 15% (5, 9) to 36.5% (6) of COVID-19 patients had a previously diagnosed hypertension. The prevalence rate varied according to the age of the included patients in each study (7), and for example, common comorbidities were hypertension (40.8%) in elderly patients (29). Based on the recently published clinical and epidemiological characteristics of COVID-19 patients (4–12), several editorials and reviews, published in famous cardiology journals, pointed to the higher risk of COVID-19 infection, the more severe disease, and augmented mortality outcomes among the infected elderly (30–32). Accordingly, it was plausible to think that the higher prevalence of hypertension in COVID-19 patients is expected and doesn't necessarily imply that hypertension is causally related to the infection with, severity of, or mortality from the new coronavirus, since hypertension is highly prevalent in the elderly (31, 33, 34). However, there was no sufficient evidence to show that subjects with hypertension are more likely to be diagnosed with the severe COVID-19 illness or proceed to a poor clinical outcome including the death due to COVID-19 than those without hypertension, independent of the age or other confounding factors.

The analysis of the clinical data of 310 COVID-19 patients (113 with and 197 without hypertension, median (IQR) of age = 62 (49, 70) years, and 56% were males) admitted to two hospitals in Wuhan, suggested a tendency to develop severe inflammation, organ damage and poor prognosis in patients with hypertension than those without hypertension. However, after adjusting for the patients' age and sex, the increased odds of hypertension in those who had the COVID-19 severe illness vs. the non-severe illness, and in those who died due to COVID-19 vs. those who were discharged alive didn't reach the significance level; ORs (95% CIs) were 1.45 (0.93–2.63) and 1.26 (0.68–2.33), respectively (6). On the other hand, a larger study that included 3,340 COVID-19 patients (1,128 with and 2,302 without hypertension, median (IQR) of age = 64 (55, 69) years, and 53% were males) admitted to nine hospitals in Hubei), the HR (95%CI) of mortality due to COVID-19 after the adjustment for age, sex, and comorbid diabetes, cerebrovascular diseases, and chronic renal disease was 1.41 (1.03–1.94) in patients with hypertension in reference to those without hypertension (18).

In our study, we found that hypertension was prevalent in 40.5% of the patients whose age was older than that reported in the previous studies and the male sex represented two-thirds of the cohort sample. Hypertension was significantly associated with both the severity of and mortality from the COVID-19 illness, even after controlling for the patients' age and sex (by adjustment or propensity score-matching); the OR of hypertension in the patients with the severe illness was 2.4- to 2.9-fold higher than that in the patients with the non-severe illness, and the HR for mortality was as twice higher in patients with hypertension as that in patients without hypertension after the adjustment of the patients' occupation, location, and the number of other underlying diseases besides, the adjustment or matching for age and sex.

The exact mechanisms by which hypertension could associate with the risk of the COVID-19 infection, its severity, and the mortality outcomes warrant further biologic and clinical investigations. However, the suggested mechanisms were mainly concentrating on the high affinity of the SARS-COV-2 to the angiotensin-converting enzyme 2 (ACE2) receptors (13, 32, 35, 36). This was shown to facilitate the viral binding to the targeted epithelial cells of the lung, heart and other organs. The debate was about how the use of the angiotensin-converting enzyme inhibitors and the ACE2 blockers drugs, commonly used in patients with hypertension would affect the risks of COVID-19 infection, severity, and mortality. Some investigators suggested beneficial effects of those drugs via not only controlling the blood pressure levels, but also reducing the inflammatory response, and blocking the viral entry to the lung and cardiac cells, while others suggested deteriorating effects through the possible retrograde feedback mechanism, by which ACE2 receptors are upregulated after being blocked by those drugs leading to increased binding sites for SARS-CoV-2 and preferential COVID-19 infection (32). However, the current protocol of COVID-19 management does not recommend patients with hypertension who are taking these drugs to stop them, because there was no significant evidence to support an association between the administration of these drugs and the higher risk for severe COVID-19 infection (4, 17). It has been also hypothesized that the hypertension-related immoderate activation of renin-angiotensin-aldosterone system (37) might motivate the NADH/NADPH oxidase system (38), prompt a massive inflammatory response and cytokine storm (39), and stimulate vascular cell contraction and constriction (40). COVID-19 patients with hypertension showed higher leukocytes count, aggressive radiological pulmonary injuries, and increased plasma levels of cytokines than patients without hypertension (6).

In our study, compared with COVID-19 patients without hypertension, patients with hypertension were more likely to have two or more chronic disease comorbidities and the most common ones were diabetes (32.3 vs. 10.7%), cardiovascular diseases (29.1 vs. 10.4%), respiratory diseases (26.4 vs. 13.0%), and cancer (26.0 vs. 13.7%). These findings were in line with those from the previous studies (4–12, 17, 37, 41). The higher prevalence of these comorbidities could add to the explanation of the higher risks of the more severe disease and mortality in patients with hypertension. A previous study indicated that the median time from COVID-19 illness onset (i.e., before admission) to death was 18.5 days (IQR, 15–22) (4); while in our study, it was shorter 17.0 (IQR, 12–22) days. The median survival time was significantly shorter in patients having those comorbidities besides hypertension than in those having these comorbidities without hypertension. This was more evident for the patients diagnosed with the severe than those diagnosed with the non-severe COVID-19 illness. However, in 113 COVID-19 patients with hypertension, Huang et al. (6) had reported no statistical difference in the laboratory tests and clinical indices between patients with other comorbidities besides the hypertension (*n* = 48) and those without any other comorbidity (*n* = 65), and suggested a limited confounding role of these comorbidities in the association of hypertension with the severity of and mortality from the COVID-19 illness.

A systematic review demonstrated that older age (≥65 years old), male gender, hypertension, cardiovascular diseases, diabetes, chronic obstructive pulmonary disease and malignancies were associated with greater risk of death from COVID-19 infection (42). Another observation emerged in our stratified analyses by the hypertension status, was the augmented risk of mortality in the hypertension group than that in the non-hypertension group for all the associated factors with the COVID-19 mortality. For example, the increased risk with the advanced age and male sex in the unmatched analysis, and the persistently increased risk in the unmatched and matched analyses in different occupations, comorbidities, and the increasing number of underlying diseases was stronger in the hypertension group than the non-hypertension group. The moderate COVID-19 illness, in reference to the mild, was associated with higher mortality in patients with hypertension but not in those without hypertension. These above-mentioned factors were previously shown to be associated with the COVID-19 mortality in general (40); however, our findings of the stratified analyses by the hypertension status weren't verified by any previous studies, although systematic reviews and editorial concluded further that hypertension could significantly increase the risks of severity and fatality of SARS-CoV-2 infection (43–45).

Our study has several limitations. Owing to its retrospective design, the urgency of time, as well as the difficulties in obtaining the data, the current study lacks important dynamic clinical and laboratory testing data. An example was the type and dosage of the used medication, nevertheless the anti-hypertension drugs. It was indicated that COVID-19 patients with hypertension who were treated with the angiotensin-converting enzyme inhibitors and the ACE2 blockers drugs were at lower risk of mortality when compared with those treated with other antihypertensive medication; the propensity score-matched and multivariable-adjusted HR was 0.30 (0.12–0.70) (18). Regarding the lack of clinical data, we could not adjust or match for the levels of factors such as C-reactive protein, creatinine, or cardiac troponins, these factors were markers for renal and cardiac injuries that were associated with the COVID-19 severity and mortality (46, 47); however, we have adjusted for the major comorbidities including cardiovascular diseases. Also, the diagnosis of hypertension was based on the medical history data, which might have led to some inevitable classification. Last, our analyses were based on a cohort of deceased COVID-19 patients; the patients' characteristics, the prevalence of hypertension, and the associations between the hypertension status and the disease severity could differ in the patients who were discharged alive after COVID-19 infection.

## Conclusion

Analyzing the data of 1,833 deceased COVID-19 patients during the early epidemic of Wuhan city, China indicated that hypertension was prevalent in over 40% of the cases, and was more prevalent in patients of the severe illness than the non-severe illness. Hypertension was associated with the increased risk of mortality in COVID-19 patients independent of the age, sex, occupation, location, comorbidities, and the number of underlying diseases. The magnitude of the associations of the demographic and clinical characteristics of the patients with the risk of mortality in COVID-19 illness (advanced age, male sex, occupation, location, COVID-19 severity, and underlying comorbidities) was higher and the median survival time was shorter in patients with hypertension than in those without hypertension.

## Data Availability Statement

The datasets processed and analyzed during the current study are available from the corresponding author on reasonable request.

## Ethics Statement

The studies involving human participants were reviewed and approved by the Ethics Committee of Medical Department of Wuhan University (WHU2020-2020YF0031). The patients/participants provided their written informed consent to participate in this study.

## Author Contributions

CY and QL collected the data. CY, JC, and SM conceptualized the design. SM did the data analysis. XL wrote the first draft of the paper. EE, KL, FW, FS, HW, and JB reviewed and provided comments on the first draft. SM and XL revised and prepared the final draft. All authors have reviewed and agree to publish the final manuscript.

## Conflict of Interest

The authors declare that the research was conducted in the absence of any commercial or financial relationships that could be construed as a potential conflict of interest.
