# Supplementary material for: The Association of Hypertension With the Severity of and Mortality From the COVID-19 in the Early Stage of the Epidemic in Wuhan, China: A Multicenter Retrospective Cohort Study
Source: Front Med (Lausanne). 2021 May 12;8:623608. doi: 10.3389/fmed.2021.623608 (PMC8149896; doi:10.3389/fmed.2021.623608)
Supplement: Supplementary file 1 [file Image_1.pdf]

frontiers

Supplementary Material

Supplementary Figure

Supplemental Figure 1. Kaplan Meier survival analysis in COVID-19 patients with and without hypertension (overall, and stratified by non-severe and severe COVID-19 infection) in total 1833 patients, and in specific accompanied comorbidities.

Chronic medical histories of patients with hypertension	Median estimate (95% CI)	Survival Curve of patients	Chronic medical histories of patients without hypertension	Median estimate (95% CI)	Survival Curve of patients
Diabetes	18 (16.53, 19.16)	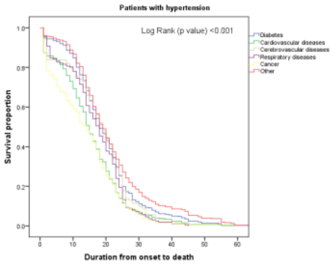	Diabetes	19 (17.67, 22.32)	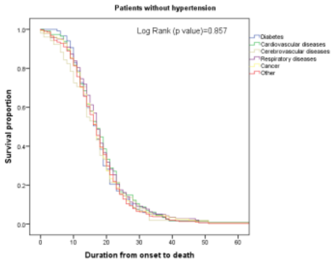
Cardiovascular diseases	15 (13.97, 16.06)		Cardiovascular diseases	18 (15.69, 19.30)	
Cerebrovascular diseases	19 (16.17, 21.22)		Cerebrovascular diseases	19 (16.69, 20.31)	
Respiratory diseases	18 (16.11, 19.88)		Respiratory diseases	21 (18.37, 22.62)	
Cancer	14 (11.73, 16.26)		Cancer	18 (17.68, 20.11)	
Other	19 (17.61, 20.38)		Other	22 (18.71, 23.28)	
Overall	17 (16.43, 17.56)		Overall	20 (19.10, 22.89)	

Chronic medical histories of Non-severe patients with hypertension	Median estimate (95% CI)	Survival Curve of patients	Chronic medical histories of Non-severe patients without hypertension	Median estimate (95% CI)	Survival Curve of patients
Diabetes	21 (19.68, 22.31)	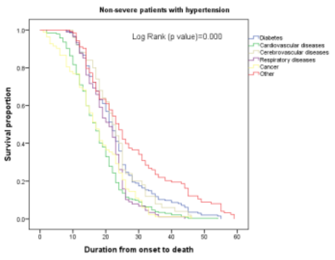	Diabetes	20 (14.01 ,20.98)	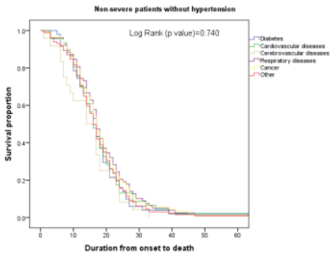
Cardiovascular diseases	16 (14.78, 17.21)		Cardiovascular diseases	20 (15.30, 19.69)	
Cerebrovascular diseases	22 (19.19, 24.80)		Cerebrovascular diseases	18 (12.93, 23.06)	
Respiratory diseases	19 (16.55, 21.44)		Respiratory diseases	20 (15.43, 21.56)	
Cancer	15 (12.29, 17.70)		Cancer	19 (13.88, 18.11)	
Other	24 (21.75, 26.24)		Other	20 (15.58, 18.41)	
Overall	19 (18.06, 19.94)		Overall	20 (16.11, 22.38)	

Chronic medical histories of Severe patients with hypertension	Median estimate (95% CI)	Survival Curve of patients	Chronic medical histories of Severe patients without hypertension	Median estimate (95% CI)	Survival Curve of patients
Diabetes	16 (14.61, 17.38)	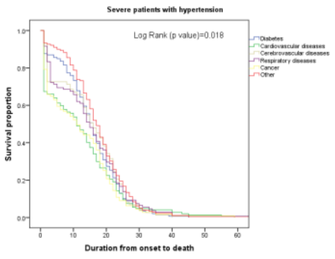	Diabetes	17 (14.61, 19.38)	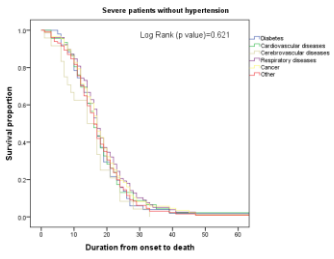
Cardiovascular diseases	11 (08.03, 13.97)		Cardiovascular diseases	16 (13.03, 18.97)	
Cerebrovascular diseases	16 (12.51, 19.48)		Cerebrovascular diseases	14 (09.51, 18.48)	
Respiratory diseases	15 (12.45, 17.54)		Respiratory diseases	17 (15.45, 18.54)	
Cancer	11 (07.02, 14.97)		Cancer	17 (15.02, 18.97)	
Other	17 (15.54, 18.45)		Other	16 (14.54, 17.45)	
Overall	15 (14.09, 15.90)		Overall	17 (16.09, 17.90)	

Supplemental Figure 1 legend

The survival curves for patients comorbid with diabetes were in blue, the green curves were for those with cardiovascular disease, the grey ones were for cerebrovascular diseases, the purple were for the respiratory diseases, the yellow were for cancer, and the red were for patients with other comorbidities.

The difference in the survival time between the two groups of COVID-19 patients (with hypertension and without hypertension) was examined using the Log rank test. The p value was (0.015) for overall, (0.075) for non-sever, and (0.014) for severe COVID-19 patients. Those for the accompanying comorbidities among patients with versus without hypertension were (0.46) for diabetes, (0.045) for cardiovascular diseases, (0.86) for cerebrovascular diseases, (0.041) for respiratory diseases, (0.021) for cancer, and (0.048) for other diseases.
